# The Clinical Course of Acute Kidney Disease after Cardiac Surgery: A Retrospective Observational Study

**DOI:** 10.1038/s41598-020-62981-1

**Published:** 2020-04-16

**Authors:** Ryo Matsuura, Masao Iwagami, Hidekazu Moriya, Takayasu Ohtake, Yoshifumi Hamasaki, Masaomi Nangaku, Kent Doi, Shuzo Kobayashi, Eisei Noiri

**Affiliations:** 1 Department of Nephrology and Endocrinology, The University of Tokyo Hospital, Tokyo, Japan; 2 Department of Hemodialysis and Apheresis, The University of Tokyo Hospital, Tokyo, Japan; 3 Department of Health Services Research, Faculty of Medicine, University of Tsukuba, Ibaraki, Japan; 4 Department of Nephrology, Immunology, and Vascular Medicine, Kidney Disease and Transplant Center, Shonan Kamakura General Hospital, Kamakura, Japan; 5 Department of Emergency and Critical Care Medicine, The University of Tokyo Hospital, Tokyo, Japan; 6 National Center Biobank Network, National Center for Global Health and Medicine, Tokyo, Japan

**Keywords:** Acute kidney injury, Interventional cardiology, Epidemiology

## Abstract

Acute kidney disease (AKD), or renal dysfunction persisting >7 days after an initiating event of acute kidney injury, is a rising concern. This study aimed to elucidate the clinical course of AKD after cardiac surgery with data on post-cardiac surgery patients admitted to intensive care units (ICU) at 18 Japanese hospitals during 2012–2014. Using multivariable logistic models, we evaluated the association of AKD with 90-day mortality and the 50% eGFR decline during 2-year follow-up compared to eGFR at 90 days. AKD was defined as an elevation in serum creatinine to at least 1.5-fold from baseline in >7 days after ICU admission. Of the 3,605 eligible patients undergoing cardiac surgery, 403 patients (11.2%) had AKD. Multivariable analysis revealed that the adjusted odds ratio (OR) of AKD for 90-day mortality was 63.0 (95% confidence interval [CI], 27.9–180.6). In addition, the adjusted OR of AKD for 50% eGFR decline was 3.56 (95% CI, 2.24–5.57) among hospital survivors. In conclusion, AKD after cardiac surgery was associated with higher 90-day mortality and renal function decline after hospital discharge.

## Introduction

It is well known that acute kidney injury (AKI) is a significant risk of developing subsequent proteinuria and chronic kidney disease (CKD)^1,2^. AKI and CKD seem to be separate conceptualized models because AKI refers to a clinical syndrome characterized by a rapid decrease in renal function while CKD refers to the presence of kidney damage or decreased kidney function for three months or more^3,4^. However, recent studies show that AKI and CKD are not always discrete and can form a continuum with patients who have a sustained renal dysfunction, having an increased risk of developing de novo CKD or deteriorating underlying CKD^5^.

Acute kidney disease (AKD), a condition in which renal dysfunction persists over seven days or more after an exposure, is a rising concern because it could play a key role in AKI to CKD transition^6–8^. The term AKD describes the clinical course of acute or subacute damage and/or loss of renal function for a duration of between 7 and 90 days after an AKI-initiating event^6,7^. It is considered that AKD is mostly a consequence of AKI in patients whose renal function is not fully recovered within seven days and progresses to CKD if the AKD is not fully recovered^6,7^. However, the trajectory of AKD remains poorly elucidated.

Cardiac surgery-associated AKI (CSA-AKI) is the second most common type of AKI (next to septic shock) and is associated with high in-hospital mortality^9,10^. In addition, CSA-AKI is proven to be a risk for CKD^11–13^. Among the characteristics of CSA-AKI are the various timings of occurrence (early or late phase after cardiac surgery) and the various clinical courses (transient or persistent renal dysfunction) due to the complex pathophysiology of CSA-AKI, which includes hemodynamic instability, mechanical stress such as cardiopulmonary bypass, inflammation, oxidative stress, neurohormonal factors, nephrotoxic agents and postoperative complications such as infections^14^. While AKI occurring early after cardiac surgery is well investigated^15–17^, AKD after cardiac surgery remains poorly studied.

Using a large Japanese database including data from ICUs at 18 hospitals, we investigated the clinical course of AKD after cardiac surgery, including its association with AKI (transient and persistent AKI), 90-day mortality, renal prognosis at hospital discharge, and long-term renal prognosis.

## Materials and Methods

### Data source and study participants

The current study is a secondary analysis of data extracted from a previous study on the AKI incidence and outcomes in the Tokushukai Medical Database^18^. The study was conducted in accordance with the Declaration of Helsinki and was approved by the Tokushukai Group Joint Ethics Committee (TGE00572-024). This committee determined that informed consent was waived because the data were anonymous.

For this study, we identified patients admitted to intensive care units (ICUs) after cardiac surgery (coronary artery bypass graft [CABG] and/or valve surgery) at 18 hospitals between 2012 and 2014. We excluded patients who died within seven days of ICU admission, because this study focused on the presence or absence of AKD after cardiac surgery. We also excluded patients who had already received renal replacement therapy (RRT).

### Definition of AKI, AKD and outcomes

**Figure 1:**
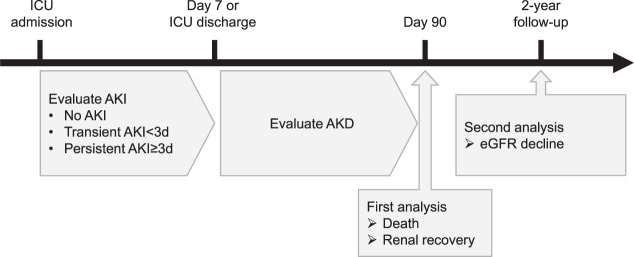
Definition of exposure and outcomes of the study. Every patient undergoing cardiac surgery was admitted to surgical ICU and evaluated for kidney function. In this study, AKI was defined as serum creatinine elevated to >0.3 mg/dL or 1.5 times higher than the baseline value within seven days after ICU admission, and AKD as an elevation in serum creatinine to at least 1.5-fold from baseline in >7 days after ICU admission. Patients were divided into three groups within seven days: those with no AKI, transient AKI (persisting for less than three days) and persistent AKI (persisting for three days or longer but less than 7). In the first analysis, AKD was evaluated for association with 90-day mortality and renal recovery from hospital discharge. In the second analysis, AKD was evaluated for association with subsequent decline of estimated glomerular filtration rate (eGFR) during the 2-year follow-up.

The definitions of AKI, AKD and relevant outcomes are briefly depicted in Fig. 1.

AKI was defined as serum creatinine value elevated to ≥ 0.3 mg/dL or 1.5 times higher than the baseline value (i.e., the most recent serum creatinine value during 6 months before hospitalization) within seven days after ICU admission. Patients with AKI were classified by AKI stage based on the worst serum creatinine value taken during seven days after ICU admission using the AKI criteria according to ‘Kidney Disease: Improving Global Outcome (KDIGO) Clinical Practice Guideline for Acute Kidney Injury’^19^. AKI was further divided into transient AKI (persisting for less than three days) and persistent AKI (persisting for three days or longer but less than).

AKD was defined as serum creatinine value elevated to 1.5 times higher than the baseline value >7 days after ICU admission. The severity of AKD was classified according to the criteria recommended by the Acute Disease Quality Initiative (ADQI) 16 workgroup^7^.

The outcomes of the study included: 90-day mortality; 90-day renal recovery, defined as the patient being alive with the serum creatinine value being recovered to less than 1.5 times the baseline value, according to the ADQI^7^; and subsequent renal function decline, defined as 30%, 40% and 50% decline (confirmed by two or more consecutive measurements separated by at least 90 days) of the estimated glomerular filtration rate (eGFR) during the 2-year follow-up, as compared to eGFR at 90 days^20,21^. These eGFR declines were selected as outcome because they are proposed as candidate surrogate markers for development to end-stage renal disease (ESRD) in previous studies. There is a controversy on what extent of eGFR decline is better so that we evaluated the association with AKD and all of candidates^22^.

### Statistical analysis

First, the baseline characteristics were compared between the three groups: patients without either AKI or AKD (No AKI), patients with AKI alone (AKI+/AKD−) and those with AKD with or without AKI (AKD+). Continuous variables were reported as means and standard deviations (SD). Categorical variables were expressed as frequencies and proportions. The intergroup differences were tested with ANOVA for age, baseline serum creatinine value and baseline eGFR (mL/min/1.73m2), and with a chi-square test for the other variables.

90-day mortality was crudely compared between the No AKI, AKI+/AKD− and AKD+ groups. Then, using a multivariable logistic regression model, we estimated an independent association between AKD and the 90-days mortality, adjusting for age, sex, AKI status, diabetes, hypertension, baseline eGFR, type of cardiac surgery, use of cardiopulmonary bypass, and non-elective surgery. Interaction between AKD and baseline eGFR was also evaluated in a univariate logistic regression model.

For the 90-days survivors, we crudely compared renal recovery, as well as the incidence of 30%, 40% and 50% eGFR decline within 2 years, among above-mentioned three groups. We also conducted multivariable logistic regression analyses, adjusting for the same covariates as the analysis for 90-day mortality. Interaction between AKD and baseline eGFR was also evaluated in a univariate logistic regression model.

Goodness of fit for these logistic regression models was tested by Hosmer-Lemeshow test for 10 groups. Multicollinearity between variables was assessed by the variance inflation factor (VIF) and no variable in the models had an associated VIF greater than 2, suggesting no multicollinearity.

Statistical analyses were conducted using R version 3.4.3 (R Development Core Team, Vienna, Austria). A conventional α level of 0.05 was used to assess statistical significance.

## Results

### Flow chart

**Figure 2:**
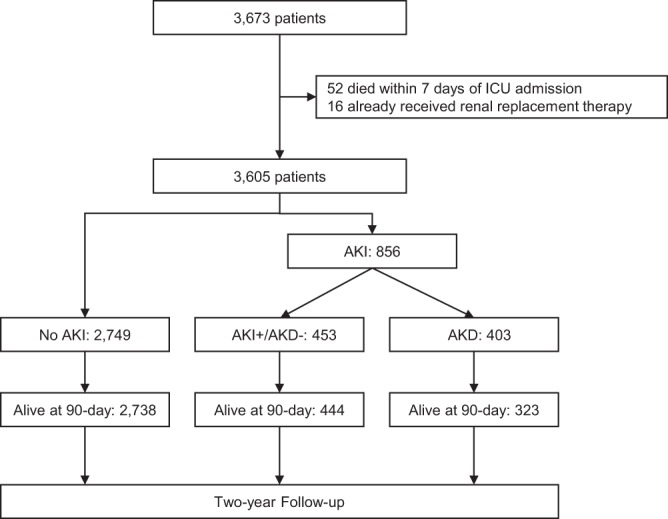
Patient flowchart.

Of the 3,673 patients admitted to ICUs after cardiac surgery, 52 patients died within seven days of ICU admission and 16 patients had already received RRT. Therefore, the remaining 3,605 patients were eligible for this study (Fig. 2).

As for transition to AKD, 38.6% of patients with transient AKI (n = 651) and 74.1% of those with persistent AKI (n = 205) progressed to AKD. Thus, 403 (11.2%) of the 3,605 eligible patients progressed to AKD. A more detailed description of the AKI/AKD stages are shown in Supplemental Figure.

### Baseline characteristics

The baseline characteristics are described in Table 1. Patients with AKD were older than no AKI patients. The proportion of those undergoing non-elective surgery in AKD patients was higher than that in other groups.

**Table 1:** Baseline characteristics of patients with or without acute kidney disease.

	No AKI (n = 2749)	AKI+/AKD− (n = 453)	AKD+ (n = 403)	*P* value
Age (years)	70.2 ± 10.9	72.2 ± 9.8	72.3 ± 11.3	<0.001
Male (n, %)	1717 (62.5%)	332 (73.3%)	260 (64.5%)	<0.001
Diabetes mellitus (n, %)	1080 (39.3%)	184 (40.6%)	156 (38.7%)	0.83
Hypertension (n, %)	2065 (75.1%)	321 (70.9%)	249 (61.8%)	<0.001
Baseline serum creatinine value (mg/dL)	0.9 ± 0.4	1.1 ± 0.5	1.1 ± 0.8	<0.001
Baseline eGFR (mL/min/1.73 m^2^)	72.5 ± 34.3	57.5 ± 33.1	62.2 ± 38.0	<0.001
CKD (n, %)*	1080 (39.3%)	263 (58.1%)	202 (50.1%)	<0.001
Type of cardiac surgery (n, %)				<0.001
CABG	1191 (43.3%)	170 (37.5%)	191 (47.4%)	
Valve	1296 (47.1%)	216 (47.7%)	162 (40.2%)	
CABG + Valve	262 (9.5%)	67 (14.8%)	50 (12.4%)	
Cardio-pulmonary bypass (n, %)	2112 (76.8%)	385 (85%)	309 (76.7%)	<0.001
Non-elective surgery (n, %)	724 (26.3%)	178 (39.3%)	250 (62.0%)	<0.001
AKI (n, %) **				<0.001
No AKI	2749 (100%)	0 (0%)	0 (0%)	
Transient AKI	0 (0%)	400 (88.3%)	251 (62.3%)	
Persistent AKI	0 (0%)	53 (11.7%)	152 (37.7%)	

AKD, acute kidney disease; AKI, acute kidney injury; CABG, coronary artery bypass grafting; CKD, chronic kidney disease; eGFR, estimated glomerular filtration rate.

*CKD was defined as the baseline eGFR (the most recent eGFR before cardiac surgery) <60 mL/min/1.73 m^2^.

**AKI was defined as serum creatinine value elevated to ≥0.3 mg/dL or 1.5 times higher than the baseline serum creatinine value (i.e., the most recent serum creatinine value before cardiac surgery) within seven days after ICU admission. AKI was further divided into transient AKI (persisting for less than three days) and persistent AKI (persisting for three days or longer but less than 7).

While baseline eGFR was associated with the occurrence of AKD among AKI patients, in a univariate logistic regression model (*P* < 0.01), receiver operation characteristic (ROC) analysis demonstrated that baseline eGFR did not predict AKD well (area under the curve [AUC], 0.57). A multivariable logistic regression analysis revealed that AKD was associated with non-elective surgery alone (Table 2) and ROC analysis demonstrated an AUC of 0.61 for non-elective surgery predicting AKD.

**Table 2:** Adjusted odds ratio for the occurrence of AKD.

Factors	Odds ratio
Age (every 10 years)	1.01 (0.87–1.16)
Male (vs. Female)	0.64 (0.47–0.88)
Diabetes (Yes vs. No)	0.9 (0.67–1.20)
Hypertension (Yes vs. No)	0.76 (0.56–1.02)
eGFR (per 10 mL/min/1.73 m^2^)	1.03 (0.99–1.07)
Type of surgery	
CABG	Reference
Valve	0.77 (0.55–1.08)
CABG + Valve	0.81 (0.51–1.28)
Cardio-pulmonary bypass (Yes vs. No)	0.69 (0.46–1.02)
Non-elective surgery (vs. elective)	2.15 (1.61–2.87)

AKD, acute kidney disease; AKI, acute kidney injury; CABG, coronary artery bypass graft.

### Outcomes of patients with and without AKD

The 90-day mortality rates were 0.4% (12/2,749), 2.0% (9/453) and 19.9% (80/403) in No AKI, AKI+/AKD− and AKD+ groups, respectively (*P* < 0.001). The 90-day mortality rates by AKI and AKD status are given in Supplemental Table. After adjustment for confounding factors, AKD was significantly associated with 90-day mortality after cardiac surgery, with an adjusted odds ratio of 63.0 (95% CI, 27.9–180.6) (Table 3). This model had adequate goodness of fit (Hosmer-Lemeshow test, χ^2^ = 7.63, *P* = 0.47). Univariate logistic regression analysis demonstrated no significant interaction between baseline eGFR and AKD for 90-day mortality (*P* = 0.33). The proportion of renal recovery was 54.8% (221/403) in AKD patients. Renal recovery by AKI status and AKD is shown in Supplemental Table.

**Table 3:** Adjusted odds ratios for 90-days mortality and renal recovery.

	Adjusted odds ratio (95% confidence interval)	
	90-day mortality	90-day renal recovery
AKI/AKD status		
AKI+/AKD−	8.43 (2.87–27.74)	Reference
AKD+	63.0 (27.9–180.6)	0.03 (0.02–0.06)
Age (every 10 years)	1.14 (0.93–1.43)	0.79 (0.67–0.93)
Male (vs. Female)	1.01 (0.64–1.60)	1.05 (0.75–1.48)
Diabetes (Yes vs. No)	0.73 (0.46–1.14)	1.12 (0.80–1.57)
Hypertension (Yes vs. No)	0.5 (0.32–0.77)	1.34 (0.96–1.88)
eGFR (per 10 mL/min/1.73 m^2^)	0.97 (0.91–1.03)	0.96 (0.92–1.00)
Type of surgery		
CABG	Reference	Reference
Valve	1.08 (0.64–1.85)	1.28 (0.86–1.90)
CABG + Valve	1.17 (0.58–2.30)	0.98 (0.59–1.66)
Cardio-pulmonary bypass (Yes vs. No)	0.99 (0.56–1.8)	1.12 (0.73–1.72)
Non-elective surgery (vs. elective)	1.68 (1.08–2.64)	0.58 (0.42–0.81)

AKD, acute kidney disease; AKI, acute kidney injury; CABG, coronary artery bypass graft.

Finally, among the 90-day survivors, the proportions of patients with 30%, 40% and 50% eGFR decline were higher in the AKD+ group than in the other groups (*P* < 0.001). Multivariable logistic regression analyses revealed that AKD was independently associated with 30%, 40% and 50% eGFR decline during the 2-year follow-up, with adjusted odds ratios of 1.79 (95% CI; 1.30–2.40), 2.62 (95% CI; 1.81–3.75) and 3.56 (95% CI; 2.24–5.57) (Table 4). These models had adequate calibration (30% decline, Hosmer-Lemeshow test, χ^2^ = 7.41, *P* = 0.49; 40% decline, χ^2^ = 6.62, *P* = 0.58; 50% decline, χ^2^ = 3.90, *P* = 0.87). Univariate logistic regression analysis revealed no significant interaction between baseline eGFR and AKD for 30% (*P* = 0.35), 40% (*P* = 0.40), and 50% eGFR decline (*P* = 0.53).

**Table 4:** Adjusted odds ratios for eGFR decline during the two-year follow-up.

	>30% eGFR decline		>40% eGFR decline		>50% eGFR decline	
	N (%)	OR (95% CI)	N (%)	OR (95% CI)	N (%)	OR (95% CI)
No AKI (n = 2,737)	313 (11.4%)	Reference	149 (5.4%)	Reference	70 (2.6%)	Reference
AKI+/AKD− (n = 444)	82 (18.5%)	1.53 (1.16–2.01)	48 (10.8%)	1.69 (1.18–2.40)	26 (5.9%)	1.75 (1.07–2.79)
AKD+ (n = 323)	68 (21.0%)	1.78 (1.30–2.40)	50 (15.4%)	2.62 (1.81–3.75)	35 (10.8%)	3.56 (2.24–5.57)

AKD, acute kidney disease; AKI, acute kidney injury; CI, confidence interval; Cr, creatinine; OR, odds ratio.

## Discussion

### Key findings and strengths

Previous studies proved that CSA-AKI is associated with long-term mortality^23–25^. This association is considered to be due to non-recovery or progressing renal dysfunction^26^. However, the causal relationship between AKI and long-term progressing renal dysfunction remains unknown. AKD is increasingly recognized as an entity bridging AKI and CKD, and thus a potential therapeutic target in the prevention of CKD and long-term risk of death^7^. Given that, to date, no published studies evaluated the clinical course of AKD, this study is the first to characterize AKD after cardiac surgery, evaluate the association between AKD and mortality and evaluate renal prognosis in terms of transition to CKD.

AKD represents a viable target for management and intervention for two reasons. First, AKD was associated with higher 90-day mortality. Although it remains unknown whether AKD is in the causal pathway for mortality, clinical care bundles for AKD – detecting the potential risk for AKD, identifying and avoiding the potential causes of AKD, and adjusting hemodynamic and volume status – may help reduce 90-day mortality. Second, AKD was associated with non-renal recovery and subsequent eGFR decline among the 90-day survivors. This finding indicates that AKD patients may be susceptible to additional kidney injury and CKD progression. Therefore, AKD patients may be at potential risk of progressing to end-stage renal disease and dialysis, which, in turn, are associated with a dramatically increased risk of cardiovascular disease^27^. Thus, care bundles to CSA-AKI is necessary especially to AKD patients.

Nephrologist follow-up of patients with AKI is recommended in the KDIGO clinical practice guidelines for AKI because of their risk of progression to CKD. However, a large number of patients in ICU have AKI so that nephrologists cannot attend to all ICU patients with AKI^28^. According to a previous study, only a minority of AKI patients actually received a nephrologist follow-up in real-world practice^29^. This points to the need for selecting AKI patients who may require nephrologist follow-up. This study clearly suggests that patients with CSA-AKI, especially those with AKD-associated complications, may be targeted for such follow-up.

For lack of consensus on its definition, AKD was defined in this study according to the recommendations in the Acute Kidney Quality Initiative (ADQI) 16 Workshop^7^ and was deemed appropriate, in that it helped identify a population at an increased risk for mortality and CKD progression, while there is room for improvement in the definition. Due to loss of muscle mass associated with reduced mobility during a prolonged hospital stay, the serum creatinine value after seven days following cardiac surgery may not have reflected actual kidney function. Further studies are required to elucidate the varying severity of AKD including kidney injury biomarkers and scoring systems^30^.

### Study limitations

This study has several limitations. First, no data were available in this study for other important confounders, such as cardiopulmonary bypass time, IABP requirements, hypotension, anemia, transfusion requirements and cross-clamp time, which might be associated with mortality, the incidence of AKI, and renal recovery^14,25^. In addition, we could not collect data on perioperative nephrotoxic agents, such as contrast agents, which may have led to delays in recovery from kidney injury. Therefore, unavailability of these data may be a major limitation of this study. Second, urine output was not included in the definition of AKD stages. Although AKD may have been better defined based on both serum creatinine and urine output, it is difficult to monitor urine output hourly in patients in late phases after cardiac surgery because most of these patients have been moved from ICU to general wards or home. Thus, the definition of AKD based on serum creatinine were deemed reasonable. Given that the criteria including urine output and serum creatinine may have altered the results, further studies are required. Third, this is a study that drew on a Japanese database. Thus, the current findings may not be readily generalizable to some other countries and races under different medical healthcare systems.

## Conclusion

AKD after cardiac surgery was associated with 90-day mortality, 90-day renal prognosis, and 2-year follow-up, suggesting that this particular clinical condition may call for closer attention.

## Supplementary information

Supplemental Figure and Table.

## Data Availability

All data generated or analyzed during this study are included in this published article.
