# Supplementary material for: The Clinical Course of Acute Kidney Disease after Cardiac Surgery: A Retrospective Observational Study
Source: Sci Rep. 2020 Apr 16;10:6490. doi: 10.1038/s41598-020-62981-1 (PMC7162986; doi:10.1038/s41598-020-62981-1)
Supplement: Supplementary file 1 — Supplemental Figure and Table. [file 41598_2020_62981_MOESM1_ESM.pdf]

# The Clinical Course of Acute Kidney Disease after Cardiac Surgery: A Retrospective Observational Study

Ryo Matsuura, M.D., Ph.D.,[1,2,6] Masao Iwagami, M.D., M.P.H., MSc., Ph.D.,[3] Hidekazu Moriya, M.D.,[4] Takayasu Ohtake, M.D., Ph.D.,[4] Yoshifumi Hamasaki, M.D., Ph.D.[1,2], Masaomi Nangaku, M.D., Ph.D.[1,2], Kent Doi, M.D., Ph.D.,[5] Shuzo Kobayashi, M.D., Ph.D.,[4] *Eisei Noiri, M.D., Ph.D.[1,2,6]

[1] Department of Nephrology and Endocrinology, The University of Tokyo Hospital, Tokyo, Japan

[2] Department of Hemodialysis and Apheresis, The University of Tokyo Hospital, Tokyo, Japan

[3] Department of Health Services Research, Faculty of Medicine, University of Tsukuba, Ibaraki, Japan

[4] Department of Nephrology, Immunology, and Vascular Medicine, Kidney Disease and Transplant Center, Shonan Kamakura General Hospital, Kamakura, Japan

[5] Department of Emergency and Critical Care Medicine, The University of Tokyo Hospital, Tokyo, Japan

[6] National Center Biobank Network, National Center for Global Health and Medicine, Tokyo, Japan

**Short Title**: **AKD after Cardiac Surgery**

**Corresponding Author**: *Eisei Noiri, Department of Nephrology and Endocrinology, The University of Tokyo Hospital, 7-3-1 Hongo, Bunkyo, Tokyo 113-8655, Japan

Phone: +81-3-3815-5411; Fax: +81-3-6451-8566; E-mail: noiri-tky@umin.ac.jp

**Supplemental Figure: Clinical course of AKI subtypes and AKD**

(a) Transient AKI

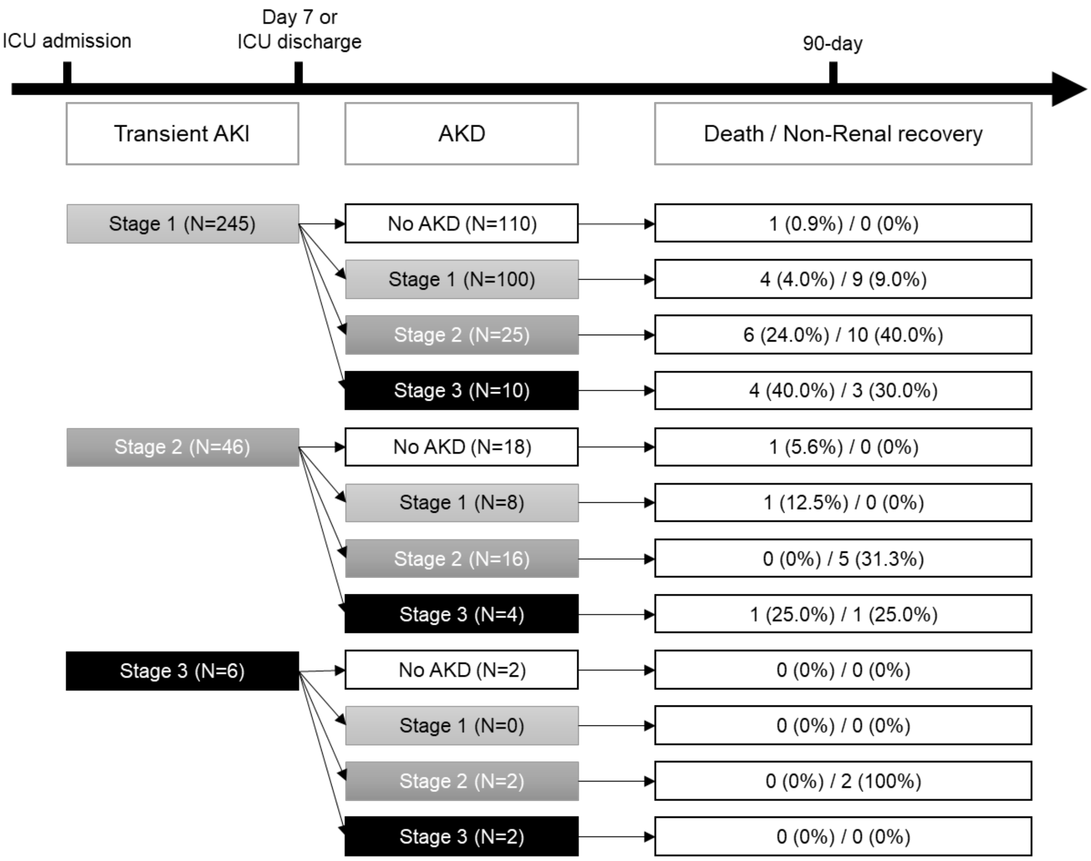


(b) Persistent AKI

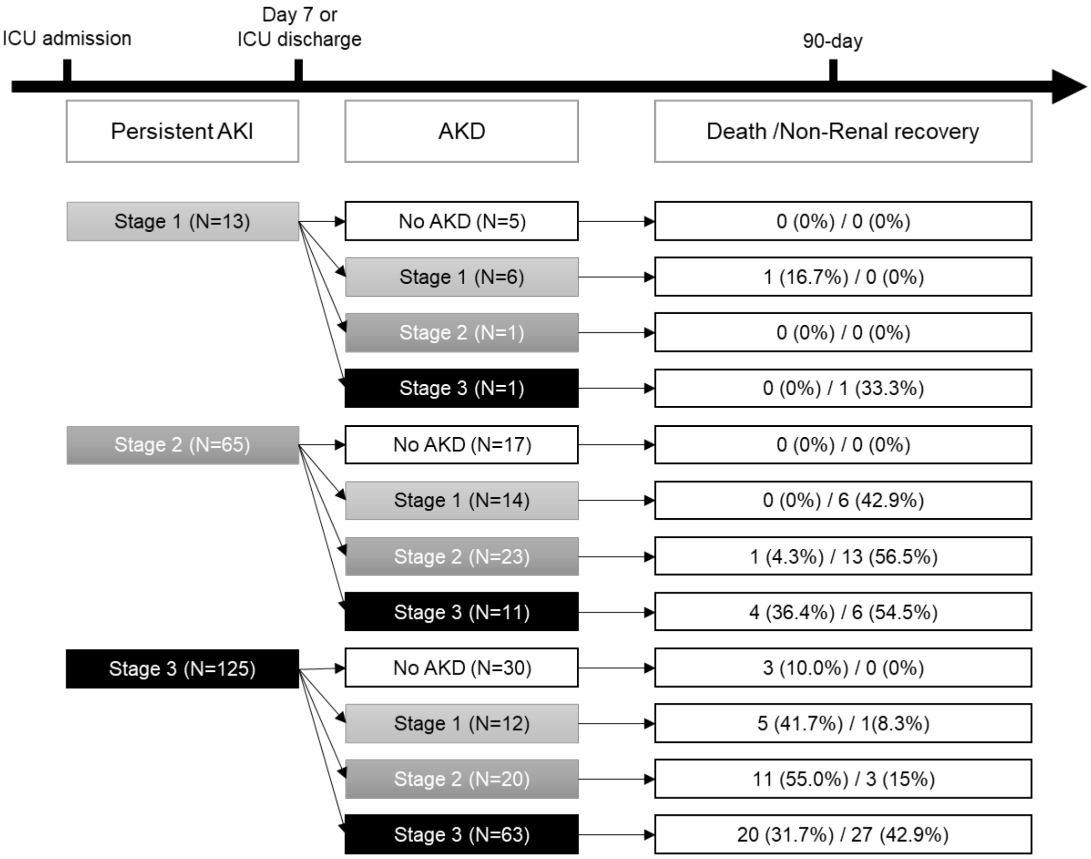

**Supplemental Table. AKI subtype, AKD, and 90-day mortality and renal recovery.**

| AKI subtype | AKD | 90-day mortality (N, %) | Renal recovery (N, %) |
|---|---|---|---|
| Transient AKI (N=651) | No AKD (N=400) | 6 (1.5%) | 392 (98.0%) |
| | AKD (N=251) | 38 (15.1%) [a] | 166 (66.1%) [a] |
| Persistent AKI (N=205) | No AKD (N=53) | 3 (5.7%) | 48 (90.6%) |
| | AKD (N=152) | 42 (27.6%) [a,b] | 56 (36.8%) [a,b] |

a, p<0.01 when compared with No AKD in any AKI subtype

b, p<0.01 when compared with transient AKI in AKD
